# Yield Gap, Indigenous Nutrient Supply and Nutrient Use Efficiency for Maize in China

**DOI:** 10.1371/journal.pone.0140767

**Published:** 2015-10-20

**Authors:** Xinpeng Xu, Xiaoyan Liu, Ping He, Adrian M. Johnston, Shicheng Zhao, Shaojun Qiu, Wei Zhou

**Affiliations:** 1 Ministry of Agriculture Key Laboratory of Plant Nutrition and Fertilizer, Institute of Agricultural Resources and Regional Planning, Chinese Academy of Agricultural Sciences (CAAS), Beijing, 100081, PR China; 2 Institute of Plant Nutrient and Resources, Beijing Academy of Agriculture and Forestry Sciences, Beijing, 100097, PR China; 3 International Plant Nutrition Institute (IPNI) China Program, CAAS-IPNI Joint Lab for Plant Nutrition Innovation Research, Beijing, 100081, PR China; 4 International Plant Nutrition Institute (IPNI), 102–411 Downey Road, Saskatoon, SK S7N4L8, Canada; Chinese Academy of Sciences, CHINA

## Abstract

Great achievements have been attained in agricultural production of China, while there are still many difficulties and challenges ahead that call for put more efforts to overcome to guarantee food security and protect environment simultaneously. Analyzing yield gap and nutrient use efficiency will help develop and inform agricultural policies and strategies to increase grain yield. On-farm datasets from 2001 to 2012 with 1,971 field experiments for maize (*Zea mays* L.) were collected in four maize agro-ecological regions of China, and the optimal management (OPT), farmers’ practice (FP), a series of nutrient omission treatments were used to analyze yield gap, nutrient use efficiency and indigenous nutrient supply by adopting meta-analysis and ANOVA analysis. Across all sites, the average yield gap between OPT and FP was 0.7 t ha^-1^, the yield response to nitrogen (N), phosphorus (P), and potassium (K) were 1.8, 1.0, and 1.2 t ha^-1^, respectively. The soil indigenous nutrient supply of N, P, and K averaged 139.9, 33.7, and 127.5 kg ha^-1^, respectively. As compared to FP, the average recovery efficiency (RE) of N, P, and K with OPT increased by percentage point of 12.2, 5.5, and 6.5, respectively. This study indicated that there would be considerable potential to further improve yield and nutrient use efficiency in China, and will help develop and inform agricultural policies and strategies, while some management measures such as soil, plant and nutrient are necessary and integrate with advanced knowledge and technologies.

## Introduction

Food security has always been a concern in China because China feeds 22% of the world’s population with 9% of the world’s arable land. The high demand for food requires an increase in crop planting area and unit yield. Maize (*Zea mays* L.) as one of the major food crops, has ranked as the most widely planted crop in China and plays an important role in securing production safety and stabilizing the grain market. While improving production should be through technological innovations to narrow the yield gap rather than area expansion[1].

Analyzing attainable yield, yield gap, nutrient use efficiency and indigenous nutrient supply is key to inform policies, prioritize research and achieve food security without environmental degradation. Attainable yield can be estimated from field or station experiments that use crop management practices designed to eliminate yield-limiting and yield-reducing factors[2,3]. While multi-plot demonstrations for several years are needed to obtain robust estimates of attainable yield to ensure that the mean estimate reflects a typical range of climatic variation[4]. The attainable yield was adopted to analyze yield gaps in this study.

Most farmers’ yields are likely to be below the yield with experimental states. The measurement and analysis of yield gaps will help improve production technologies and provide efficient targeting efforts to increase yield, because yield gap is essential to inform policies and technical research on food security[4,5]. Good nutrient management practices can narrow the yield gap to move towards maximum attainable yield[6,7]. However, the variation in yield gaps was obvious among regions, because there are great differences in climates, soil types, and crop and nutrient management practices.

Narrowing the yield gap is very important for China and chemical fertilizer plays a decisive role in reducing yield gap. However, over-fertilization by farmers driven by the desire for higher yields does not always contribute to high yield, and over or excessive fertilizer application has become a common phenomenon for farmers’ practices recently in China, particularly for nitrogen (N) and phosphorus (P) fertilizer[8], which has led to nutrient accumulation in the soil, low nutrient use efficiency and environmental pollution[9–11]. Knowing soil nutrient condition is the premise of the optimized fertilization. Soil indigenous nutrient supply can reflect the soil nutrient condition or soil fertility and can be developed as guideline for fertilizer recommendation. The higher indigenous nutrient supply means the higher grain yield in the nutrient omission plots[12]. A high soil indigenous nutrient supply is a potential threat to the environment[13,14] and must be taken into consideration when developing fertilizer recommendations[15,16], because these nutrients can seep into groundwater and pollute rivers and lakes through leaching and runoff.

Nutrient use efficiency is a direct measure for the rationality and advancement of fertilization. Some terms were frequently used in agronomic research to assess the efficiency of applied fertilizer, such as apparent recovery efficiency (RE, kg nutrient uptake increase per kg nutrient applied), agronomic efficiency (AE, kg yield increase per kg nutrient applied), partial factor productivity (PFP, kg yield per kg nutrient applied)[17–19]. Nutrient use efficiency was affected by grain yield, soil indigenous nutrient supply, amount of fertilizer application, and the overall timeliness and quality of other crop management operations[18]. Fertilizer recommendation strategies should be balanced with regard to achieving high nutrient use efficiency as well as maximizing the crop yield.

It is necessary to obtain and understand the attainable yield, yield gap and nutrient use efficiency to provide scientific basis for nutrient management in the major maize production regions of China [20–23]. Therefore, the objectives of this study were: (1) to quantify the maize yield gaps; (2) to analyze the indigenous nutrients supply and yield responses; (3) to evaluate AE, PFP, RE of optimal nutrient management (OPT) and farmers’ practices (FP) for maize.

## Materials and Methods

### Site characteristics and data source

**Fig 1:**
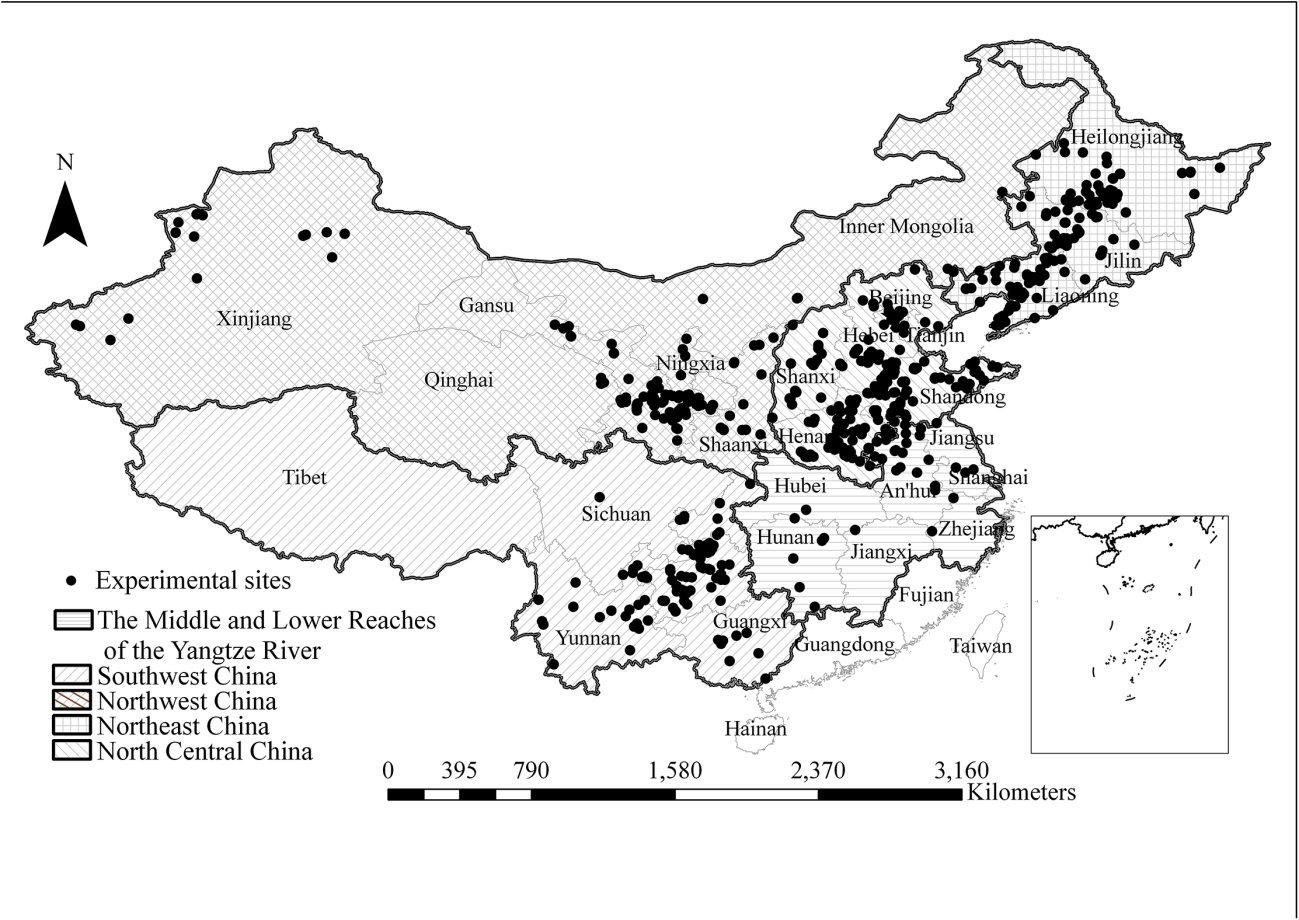
Geographical distribution of the studied locations in the Northeast (NE), North-Central (NC), Northwest (NW), Southwest (SW) and the Middle and Lower Reaches of the Yangtze River (MLYR) of China.

The field experiments used for this study included spring maize and summer maize. Spring maize is mainly planted in the Northeast (NE), Northwest (NW), and Southwest (SW) of China, while summer maize is mainly planted in North-Central (NC) China and Middle and Lower reaches of the Yangtze River (MLYR). Because there was few experiments distributed in the MLYR, and mainly conducted in An’hui and Jiangsu provinces with a rotation of winter wheat-summer maize similar to the NC region, so these data were combined into the NC region. The database used in this analysis totaled 1,971 field experiments were obtained from published and unpublished studies conducted from 2000 to 2012. The unpublished studies were conducted by the International Plant Nutrient Institute (IPNI) China Program and our research group. Relevant articles published in journals were found through the Web of Science database. The field experiments covered a wide range of soils, climatic conditions, agronomic practices and different treatments including OPT, FP and omission of N, P and K nutrients, which were described by our previous published papers[12,24]. The distribution of experimental sites was shown in Fig 1.

### Data Analysis and Quantification

Attainable yield (YA) in this study is defined as the maximum actual yield derived in the field of research station experiments under the best or optimal nutrient management practices, using up-to-date information technology and advanced management techniques. The yield gap is defined as yield difference between attainable yield and average farmers’ yield (YF). Indigenous nutrient supply is defined as the total amount of a particular nutrient that is available to the crop from the soil during a cropping cycle, when other nutrients are non-limiting[25], and can be measured in nutrient omission plots[26]. Indigenous N supply (INS), indigenous P supply (IPS), and indigenous K supply (IKS) in the current study were plant N, P, and K accumulation in the above-ground dry matter at harvest in the N, P, and K omission plots, respectively.

Yield response (YR) is another effective and immediate index of basic soil fertility, and yield responses to fertilizer N, P, and K are defined as the yield gap between attainable yield and the yield from omission plots when one of nutrient is omitted. The yield response is related to indigenous nutrient supply, which determines the yield in omission plots[15,26]. YR can be used to evaluate the soil nutrient supply capacity[12].

The nutrient use efficiency parameters including RE, AE, and PFP can be estimated from the following equations:

AEi=(Y–Y0)/Fi (1)

REi=(U–U0)/Fi (2)

PFPi=Y/Fi (3)

Where i represents N, P, or K; F is the amount of fertilizer applied (kg ha^-1^); Y is the yield with OPT or FP (kg ha^-1^); Y0 is the yield (kg ha^-1^) in a control treatment with no N, P, or K; U is the total plant nutrient uptake in aboveground biomass at maturity in a OPT or FP plot (kg ha^-1^); U0 is total plant nutrient uptake in aboveground biomass at maturity in a plot with no N, P, or K (kg ha^-1^).

The SAS V8 software was used to analyze the means of AE, RE, PFP between OPT and FP by using least significant difference at the 0.05 probability level whenever a significant F test was observed in the ANOVA. Meta-analysis was used by using Revman 5.0 software (developed by the Cochrane Collaboration, Oxford, UK) not only in order to analyze the difference between attainable yield and farmers’ yield, and between attainable yield and control plots, but also analyze the differences among regions. For each region, the weighted mean differences in yield were computed (including YA vs. YF, and yield from the N, P, or K omission plots).The Cochran Q test was used to test for heterogeneity. A *P* value <0.1 by the Cochran Q test indicated statistically significant heterogeneity. The test for overall effect for yield gap and yield response at 0.05 probability level, estimates of effect size were considered to be significantly different from zero if their 95% CIs (Confidence Intervals) did not overlap with zero[19].

## Results

### Overview of yield and yield gaps with regions

**Fig 2:**
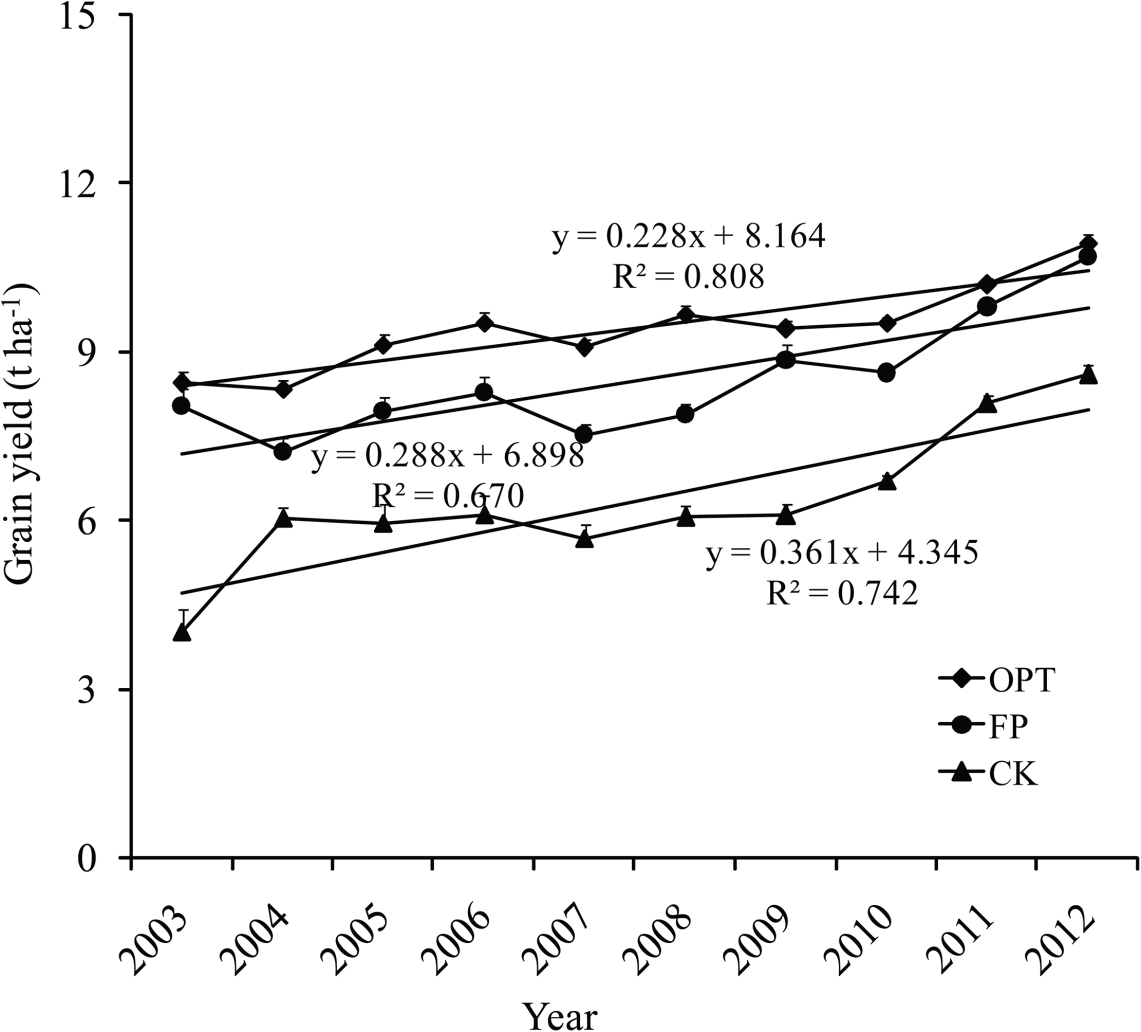
Trend in grain yield for optimal management (OPT), farmers’ practice (FP), and control treatment without fertilization (CK) in the past decade across in maize grown regions in China. Yield data come from experiments collected by our research group from 2003 to 2012, error bars represent standard error.

The highest yield was found in NE, followed by NW>NC>SW (Table 1). There was the long growth period in NE and NW than NC and SW (more than 40–50 days), and the higher day/night temperature difference also help accumulate dry matter. The meta-analysis showed that there was significant difference between YA and YF (0.7 t ha^-1^ or 8.4% across all sites, *P*<0.00001).However, the yield gap between YA and YF was different among regions (*P* = 0.07). The yield gap in the NW (1.8 t ha^-1^) was significant higher than other three regions, and large variation was existed for the 95% confidence intervals, because the technology of mulching at planting and drip irrigation were used in the OPT treatment in the NW region, which helped increase yield. However, as some agricultural technologies and equipments continue to be adopted by farmers, YF has gradually increased over the past decade and moved closer to the YA, resulting in a lower YGF (Fig 2).

**Table 1:** Yield gaps between attainable yield (YA) and farmers’ yield (YF) in experimental plots and 95% confidence interval (CI) in different production regions of China.

	YA (t ha^-1^)			YF (t ha^-1^)				Mean difference
Region^a^	Mean	SD^b^	n	Mean	SD	n	Weight (%)	IV^d^, Random, 95%CI
NE	10.8	1.9	653	10.2	1.9	290	32.9	0.6 (0.3, 0.9)^c^
NW	10.5	2.9	192	8.7	3.0	50	2.8	1.8 (0.9, 2.7)
NC	8.9	1.9	992	8.3	1.9	544	61.1	0.6 (0.4, 0.8)
SW	8.4	1.9	134	7.4	2.0	24	3.2	1.0 (0.1, 1.9)
Total (95% CI)	9.6	2.2	1971	8.9	2.2	908	100	0.7 (0.4, 1.1)
Heterogeneity	*P* = 0.07							
Test for overall effect	*P*<0.00001							

^a^NE = Northeast; NW = Northwest; NC = North-Central; SW = Southwest

^b^SD = standard deviation

^c^ 95% confidence interval in parentheses

^d^ IV = inverse variance, is pre-calculated estimate of treatment effect and standard error of estimate

### Yield response and indigenous nutrient supply

The high fertilization application rate has led to nutrient accumulation in the soil for decades, and this has resulted in the yield increased under zero N, P and K plots since the 2000 (Fig 2).The results showed that the average yield response to N, P, and K were 1.8, 1.0, and 1.2 t ha^-1^across all regions, respectively, and meta-analysis indicated that there were significant differences in YR to N (*P*<0.0001), P (*P* = 0.02), and K (*P* = 0.04) across four regions (Table 2). The highest YR to N, P, and K were observed in SW (2.5, 1.7 and 1.6 t ha^-1^, respectively), while the lowest YR to N and P were achieved in NC (1.5 and 0.9 t ha^-1^) and the lowest yield response to K in NW (0.3 t ha^-1^).

**Table 2:** Yield responses to applied N, P, and K for maize in different production regions of China.

	N (t ha^-1^)		P (t ha^-1^)		K (t ha^-1^)	
Region^a^	Yield response	n	Yield response	n	Yield response	n
NC	1.5 (1.3, 1.7)^b^	639	0.9 (0.7, 1.1)	546	1.2 (1.0, 1.4)	610
NE	1.9 (1.7, 2.2)	348	0.9 (0.7, 1.2)	290	1.2 (1.0, 1.5)	361
NW	2.4 (1.8, 3.0)	127	1.2 (0.5, 1.8)	105	0.3 (-0.4, 1.0)	94
SW	2.5 (2.1, 3.0)	100	1.7 (1.2, 2.2)	98	1.6 (1.1, 2.1)	106
Total (95% CI)	1.8 (1.6, 1.9)	1214	1.0 (0.8, 1.1)	1039	1.2 (1.1, 1.3)	1171
Heterogeneity	*P*<0.0001		*P* = 0.02		*P* = 0.04	
Test for overall effect	*P*<0.00001		*P*<0.00001		*P*<0.00001	

^a^ NE = Northeast; NW = Northwest; NC = North-Central China; SW = Southwest

^b^ 95% confidence interval in parentheses.

**Fig 3:**
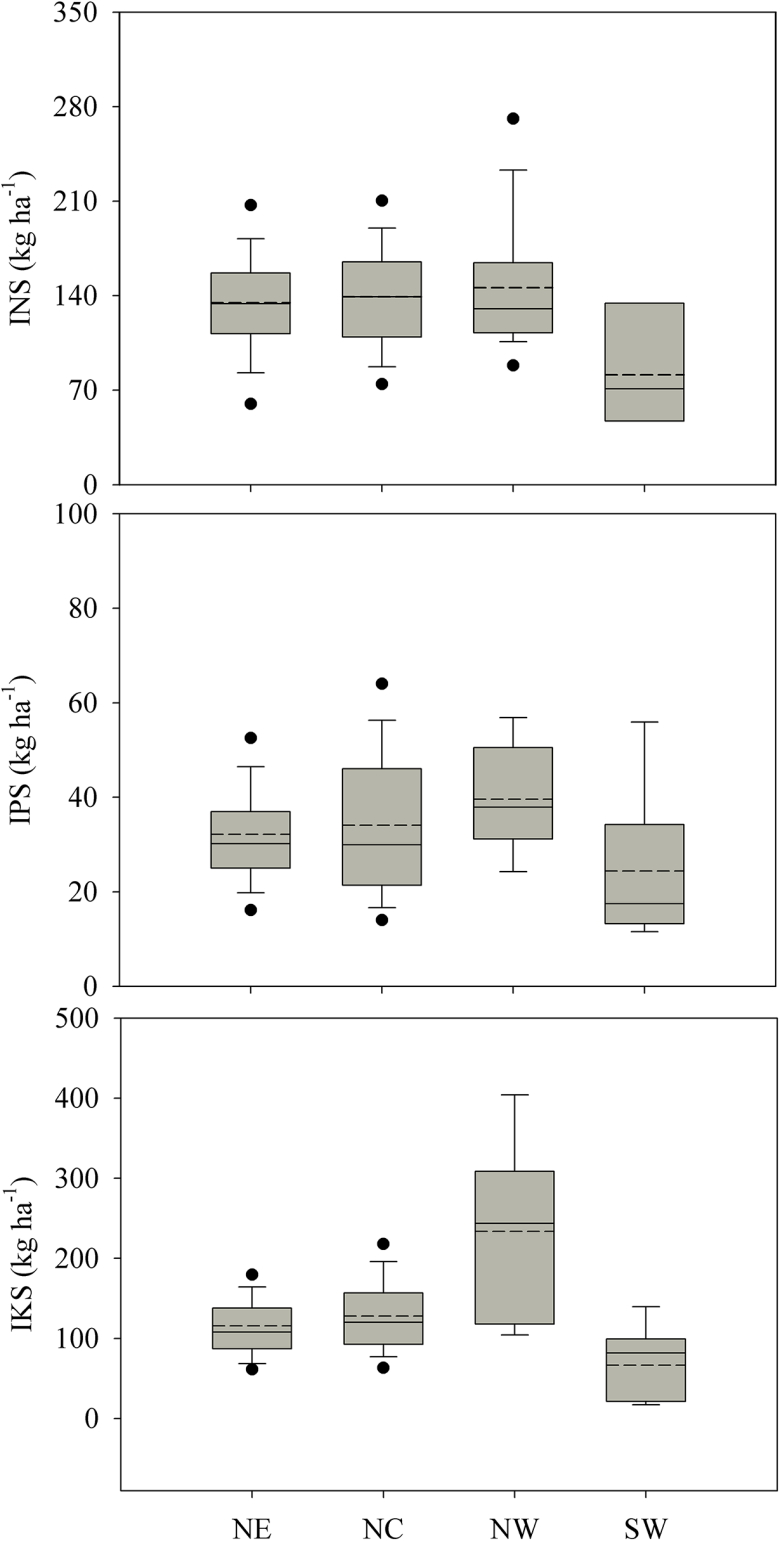
Variation in the indigenous nutrient supply for maize in Northeast (NE), North-Central China (NC), Northwest (NW) and Southwest (SW); INS, IPS, and IKS indicate indigenous N, P, and K nutrients supply, respectively. Solid and dashed lines indicate median and mean, respectively. The box boundaries indicate the upper and lower quartiles, the whisker caps indicate the 90^th^ and 10^th^ percentiles, and the circles indicate the 95^th^ and 5^th^ percentiles. Numbers in brackets denote the number of observations.

There were significant negative correlation between yield response and indigenous nutrient supply for N (*P*<0.001), P (*P* = 0.017), and K (*P*<0.001), respectively. The result (Fig 3) indicated that the difference in INS was observed among regions (*P* = 0.0134). INS in the NE (134.8 kg ha^-1^), NC (139.2 kg ha^-1^), and NW (145.9 kg ha^-1^) were significantly higher than in the SW (81.5 kg ha^-1^), there were the lowest YA (Table 1) and the highest YR to N (Table 2) in SW. There was the higher IPS in NC and NW than SW region (*P* = 0.0242). In the NW, IKS (233.7 kg ha^-1^) has about quadruple of that in the SW (66.6 kg ha^-1^), and about double of that in the NE (115.8 kg ha^-1^) and NC (128.0 kg ha^-1^) (Fig 3). This high IKS was related to the low rainfall and thus low weathering from water and low K leaching and K-bearing hydromicas with high soil K supplying capacity in NW[19]. The difference in indigenous nutrient supply among regions was also due to great variation in fertilizer application. In the NC, For example, only N fertilizer but no P and K fertilizer were applied, while in the NE, N and P were applied but no K fertilizer was applied by farmers.

### Variation in nutrient use efficiencies with management

**Fig 4:**
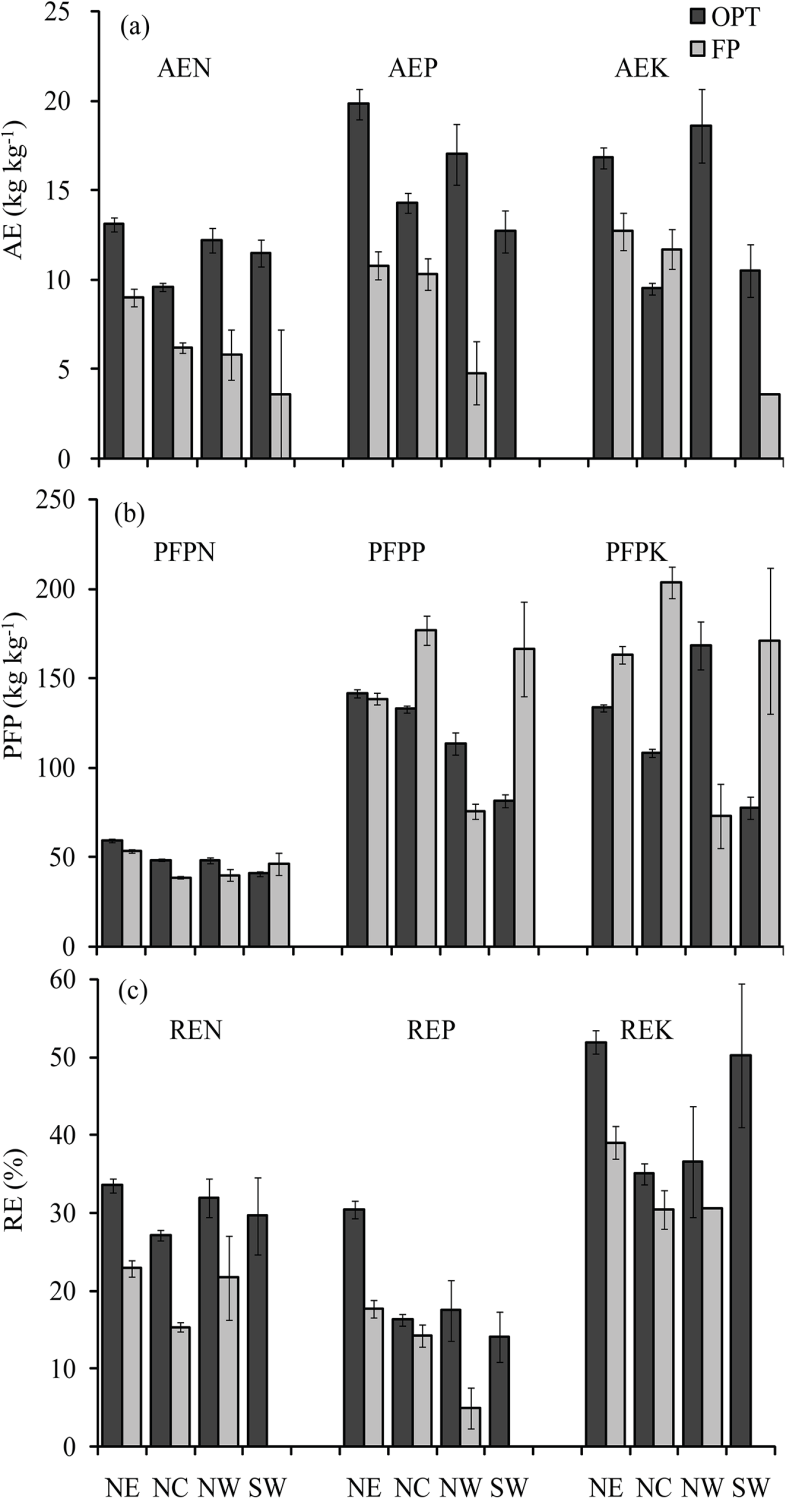
Descriptive statistics for agronomic efficiency (AE), partial factor productivity (PFP) and recovery efficiency (RE) in the optimal treatment (OPT) and farmers’ practices (FP) for maize in different production regions of China. Error bars represent standard error.

Across all regions, average AE of N and P were significantly higher (3.9 and 5.8 kg kg^-1^) in OPT than in FP, but a smaller gap for AE of K (*P* = 0.2823) (Fig 4A); The PFP of N in OPT (51.6 kg kg^-1^) was significantly greater than in FP (43.3 kg kg^-1^, n = 884) (Fig 4B), while the PFP of P and K with OPT were lower than those with FP, decreased by 23.0 and 62.5 kg kg^-1^, respectively; The average RE of N, P, and K with OPT were significantly higher than with FP, increased by percentage point of 12.2, 5.5, and 6.5, respectively (Fig 4C).

The differences in nutrient use efficiencies existed between OPT and FP among regions. The high N fertilizer application rate and unreasonable management (for instance, one time fertilizer application) were the major reasons for the low N use efficiencies for FP. There was accounting for 79.8% of all sites with fertilizer N application had higher than 180 kg N ha^-1^ for FP. Low P fertilizer application in some regions resulted in the high PFP of P, for example, about 52.7% of famers who applied P fertilizer less than 40 kg P_2_O_5_ ha^-1^ in NC. Low K fertilizer application also led to the AE of K for FP being close to the OPT (Fig 4A) and had the high PFP of K (Fig 4B), accounting for 55.1% of all sites with K fertilizer application less than 45 kg K_2_O ha^-1^ in FP, but the high RE of P and K were attained with OPT compared with FP.

## Discussion

Quantifying the attainable yield and yield gap are essential to research and formulate policies for food security and environment protection at regional and national, even international levels[5,27]. The decreasing yield gap with years (Fig 2) was mainly due to the advancement of techniques and the higher fertilizer application used by farmers, which led to higher farmers’ yield and yield without any fertilizer (Fig 2). Narrowing yield gap between YA and YF is very important to food security, while some factors led to yield losses in YF compared with YA, because YA was obtained from field of station experiments, which followed optimized management practices such as new crop variety, advanced cultivation technology, weed, pests, and disease control, while these were not adopted by farmers. Besides, yield gaps were significantly correlated with irrigation, accessibility, market influence, agricultural labor, and slope[28].

Although maize grown with OPT and FP achieved the high yield in this study, there was substantial gap as compared to some researches. For example, YF and YA yields in our study only achieved 59% and 64% of simulated potential yield (15.1 t ha^-1^) from Chen et al study [22], respectively. Approach designed for greater synchrony between crop demand for nutrients and its supply from soil, environment and applied inputs can greatly improve grain yield[6,22]. The differences in climate, soil and rotation resulted in great variation in attainable yield across regions in China. In addition, sowing date, seeding rates, transplanting patterns, water and crop management also made a significant difference in actual yield, therefore yield gaps varied across different regions[27]. If all those measures were well implemented, yield gap between YA and YF would be further narrowed.

Chemical fertilizer application tended to narrow the yield gap. However, the high fertilization also led to high soil indigenous nutrient supply[6,9,14], and thus the INS and IPS in this study were obviously higher than that previously reported by Liu et al. [29] using data from 1985 to 1995 in China (INS 75.9 kg ha^-1^, IPS 16.4 kg ha^-1^).The high INS and IPS observed in the current study was caused by long-term excessive fertilizer application in the previous crops[13,19,30]. The INS and IPS accounted for 73.1% and 84.4% of N and P uptake in the above aground dry matter, respectively.

It is necessary to maintain crop yield, minimize fertilizer loss to the environment, and optimize nutrient use efficiency in agricultural production. Nutrient use efficiency have long been the focus of academic research, government and community, and many agronomic indices have been presented to assess nutrient use efficiency[18,31]. Excessive or imbalanced fertilization (i.e., one fertilizer application) were very common by farmer’s practices, the low N use efficiencies were prevalent in China[32].The mean of RE and AE of N in the OPT experiments were still less than that measured from other studies[18,29,33].To meet cereal demand in 2025 at a modest pace of increased N consumption, the global PFP of N in cereals needs to increase at a rate of 0.1–0.4% per year[34]. Many studies have demonstrated that the farms with new N management technologies could increase REN by 30%–50%in many crops[35,36].

Compared with FP, high P and K use efficiencies were obtained with OPT, the average REP (21.2%) and REK (40.8%) for OPT in this study were close to the baseline recommended by Dobermann[18]. Management of P and K has been described for maize in many reports[32,37]. Soil testing and crop-based approaches have been developed and widely used in P and K management guidance for farmers[12,14,38]. While P and K fertilizer application were imbalanced and nutrient use efficiency were lower for famers in China. For example, the higher P fertilizer application in the NE, and very low application in the NC, while the K fertilizer application was generally lower for FP in the four regions. The K input-output budget was highly negative in regions like NE and NW, because crop straw was difficult to decompose due to low temperature in winter time[39]. The profitable and sustainable use of the soil-plant system is the basic requirements for optimum P and K management. Therefore, other agronomic strategies such as rotations, cultivations, water management, etc, also need to be integrated into nutrient management practices to guarantee high yield and nutrient use efficiency, and promote the sustainable development of the agriculture in China.

There was still more room to increase maize yield and nutrient use efficiency in China. While many factors such as the heterogeneity in the indigenous nutrients supply, yield gap and nutrient use efficiency with in smallholder farms are all essential parts in building the best management practices to sustainably increase maize yield, financial return, nutrient use efficiency and protect environment. Meanwhile, farmer knowledge, farmer learning by attending seminar, field experiment demonstration, and the existing social capital are necessary for ensuring adoption and spread of best management practices. Therefore, it is important to get a balance among economic viability, environmental health, farmer acceptance, and political infrastructure, which may be a difficult and long process for small-scale farmers in China, but, it is promising.

## Abbreviations

AE: agronomic efficiency
FP: farmers’ practice
IKS: indigenous potassium supply
INS: indigenous nitrogen supply
IPS: indigenous phosphorus supply
OPT: optimal management
PFP: partial factor productivity
RE: recovery efficiency
